# Development and characterization of nuclear microsatellite markers for *Eremanthus erythropappus* and their transferability across related species

**DOI:** 10.1186/s40659-020-00298-z

**Published:** 2020-07-07

**Authors:** Lucas Fernandes Rocha, Natália Ribeiro Paula, Alison Gonçalves Nazareno, Dulcinéia de Carvalho

**Affiliations:** 1 Department of Forest Science, São Paulo State University, UNESP, Botucatu, São Paulo state 18.610-034 Brazil; 2 Department of Forest Sciences, Laboratory of Tree Genetic Conservation, Federal University of Lavras, UFLA, Lavras, Minas Gerais state 37.200-000 Brazil; 3 Department of Biology, Federal University of Minas Gerais, UFMG, Belo Horizonte, Minas Gerais state 31.270-901 Brazil

**Keywords:** *Eremanthus erythropappus*, Simple sequence repeats, Genetic diversity, *Eremanthus incanus*, *Eremanthus glomerulatus*

## Abstract

**Background:**

We developed simple sequence repeats (SSR) for *Eremanthus erythropappus* (DC.) MacLeish, an endangered tree species endemic to the Brazilian Savanna and Atlantic Forest biomes, and tested their transferability to two closely related *Eremanthus* species.

**Results:**

Using a genomic library enriched with tandem repeat motifs, we identified 16 primer pairs, and characterized them in two populations. Nine primers amplified the expected size fragments and seven SSRs were polymorphic, providing a total of 38 alleles and an average of 4.22 alleles per marker. The polymorphic information content (PIC) ranged from 0.44 to 0.94 with an average of 0.65. The average observed heterozygosity across all loci varied from 0.61 to 1.00. The observed (*H*_*O*_) and expected (*H*_*E*_) heterozygosity within the two populations varied from 0.65 to 1.00 and from 0.31 to 1.00, respectively.

**Conclusions:**

These newly developed SSR markers are a powerful tool for population genetic analyses and may be useful in studies on species ecology, evolution, and taxonomy.

## Background

*Eremanthus erythropappus* (DC.) MacLeish is an endemic tree species native to the high-elevation mountains of the Brazilian Savanna and Atlantic Forest biomes [12, 13]. Regenerants of the *Eremanthus* genus are light-demanding, as is common with pioneer species [3, 21], and *E. erythropappus* normally occurs in shallow, rocky soils with low fertility [17]. Historically in Brazil, the species has been over harvested for use as fence posts in rural areas due to the durability of the wood against weathering [22, 23], and for the extraction of a sesquiterpene alcohol, α-bisabolol, which is used in many pharmaceutical products because of its anti-inflammatory, antibacterial, skin-smoothing, and wound healing properties [10, 24].

Microsatellite markers, also known as simple sequence repeats (SSR), are often used to study the genetic diversity of plants and animals. SSR sequences are distributed throughout the euchromatin and eukaryote genome [11, 19] and exhibit high polymorphism and repeatability across laboratories [28]. Microsatellite loci present the highest information content among all classes of molecular markers [27]. These markers can be analyzed using polymerase chain reaction (PCR), allowing for a highly informative evaluation of a large number of loci, as well as assessing effects related to species population genetics, breeding programs, and germplasm conservation [18]. The objective of this study was to develop a set of microsatellite markers to be used to evaluate the genetic diversity and structure of *E. erythropappus* and other species of the *Eremanthus* genus.

## Results

From the initial 16 primer pairs, nine amplified the expected size fragment while the remaining seven either did not amplify or generated multiple bands indicating non-specific amplification. The optimum annealing temperature was 53 °C for all nine primers. Of the nine amplified primers, seven were polymorphic (Ere02, Ere03, Ere07, Ere08, Ere10, Ere13, and Ere14) and two were monomorphic (Ere04 and Ere09). The observed and expected heterozygosities ranged from 0.31 to 1.00 and from 0.38 to 0.91, respectively. The total number of alleles was 38 and the number of alleles per polymorphic marker ranged from 2 to 18 (Table 1). The mean within-population inbreeding coefficient (*F*_*IS*_) was − 0.23. The polymorphic information content (PIC) ranged from 0.44 to 0.94, with an average of 0.65. The combined exclusion probability is sufficient to perform a paternity/maternity exclusion analysis among breeding populations using the seven polymorphic loci. Thus, for the first parent, the exclusion probability reached 0.98 and for the second parent it reached 0.99. None of the tested loci showed significant deviation from Hardy–Weinberg equilibrium, nor did they exhibit a high frequency of null alleles ($\overline{r}_{d }$ < 0.5; P > 0.05). Six SSRs (Ere03, Ere07, Ere08, Ere09, Ere13, and Ere14) cross amplified in *E. incanus* and *E. glomerulatus*.

**Table 1:** Genetic characterization of the nine developed microsatellites for *Eremanthus erythropappus* and its transferability to *Eremanthus incanus* and *Eremanthus glomerulatus*

Locus	*PIC*	Itamonte (n = 21)				Lavras (n = 21)				Transferability	
		*Na*	*H*_*O*_	*H*_*E*_	*F*_*IS*_	*Na*	*H*_*O*_	*H*_*E*_	*F*_*IS*_	*E. incanus*	*E. glomerulatus*
Ere02	0.61	3	1.00	0.56	− 0.78	3	1.00	0.61	− 0.63	–	–
Ere03	0.68	5	1.00	0.60	− 0.67	9	0.65	0.56	− 0.16	+	+
Ere04	0.44	1	0.00	0.00	0.00	1	0.00	0.00	0.00	–	–
Ere07	0.53	2	0.85	0.49	− 0.74	2	0.83	0.49	− 0.71	+	+
Ere08	0.67	2	0.95	0.49	− 0.92	3	0.31	0.38	0.19	+	+
Ere09	0.51	1	0.00	0.00	0.00	1	0.00	0.00	0.00	+	+
Ere10	0.94	11	0.84	0.84	0.27	18	0.81	0.91	0.11	–	–
Ere13	0.79	3	0.48	0.48	1.00	5	0.00	0.64	1.00	+	+
Ere14	0.67	3	0.58	0.58	− 0.54	3	0.62	0.43	− 0.45	+	+

Geographic coordinates for the populations are Itamonte = 22°16′45.00″S 44°46′25.60″W and Lavras = 21°19′51.85″S 44°57′54.76″W; All two populations are located in the Minas Gerais state, Brazil

*PIC* Polymorphic content information, *Na* Number of alleles, *Ho* observed heterozygosity; *He* expected heterozygosity, *F*_*IS*_ Inbreeding coefficient; +, successful PCR amplification; −, unsuccessful PCR amplification

## Discussion

The development of SSR markers for *E. erythropappus* enables the application of new genetic research into this endemic and overexploited tree species. Whereas *E. erythropappus* presents relevant economic interest and remarkable ecological importance, new studies will enable us to analyze the genetic diversity, gene flow and also possible processes of inbreeding and clonality. Overall, the genetic diversity estimates found herein were considerably higher than those found in previous studies on the species using inter-simple sequence repeat (ISSR) markers [6, 7, 15].

Although SSR markers are sometimes identified as monomorphic through agarose electrophoresis, they could be polymorphic considering the resolution of capillary electrophoresis (CE). While MetaPhor^TM^ Agarose (4 bp) offers a high resolution, CE presents a better resolution and greater separation efficiency (2 bp) [25]. Thus, the two monomorphic primers developed herein may also be valuable for studying genetic parameters [14]. The evidence of previous researches suggests that the high information content found in SSR markers may enable their use as primers.

## Conclusions

We developed nine microsatellite markers, of which seven are polymorphic and two are monomorphic. These markers can inform new research on population genetics, genetic diversity, spatial genetic distribution, as well as the sustainability of forest management practices employed for this species. Additionally, these primer pairs may be an important tool to assist breeding programs of this species. The analysis of cross-amplification confirmed the possibility of applying these markers in genetic studies of *E. incanus* and *E. glomerulatus*.

## Methods

We used a (GA)_n_ and (CA)_n_ microsatellite-enriched library based on Billotte et al. [1]. As such, leaf tissue samples from *E. erythropappus* adult trees were collected and preserved in silica gel. Total genomic DNA was extracted using the CTAB method according to Doyle and Doyle [4]. Thirty ng of genomic DNA was digested using the *Rsa*I restriction enzyme (Promega, Madison, Wisconsin, USA), and fragments were ligated to adapters (*Rsa*21 5′-CTCTTGCTTACGCGTGGACTA-3′ and *Rsa*25 5′-TAGTCCACGCGTAAGCAAGAGCACA-3′). For the enrichment of SSRs sequences, we used (CT)_8_, (GT)_8_, and (TTC)_8_ repeats using biotinylated microsatellite probes, and the target fragments were captured by the use of streptavidin-coated magnetic beads (Promega Corporation, Madison, Wisconsin, USA). The *Rsa*21 and *Rsa*25 adapter sequences were used as primer templates for the amplification of fragments. The microsatellite fragments were ligated to a pGEM-T Easy Vector System (Promega Corporation, Madison, Wisconsin, USA). The plasmids were introduced into *Escherichia coli* XL1-Blue strains, and transformed cells were plated on Petri dishes with Luria-Bertani (LB) agar medium containing ampicillin (100 μg ml^−1^) and X-galactosidase (5-bromo-4-chloro-indolyl-β-d-galactoside) (50 μg ml^−1^). The recombinant colonies were sequenced using an ABI 377 automated sequencer and the Big Dye Terminator Kit (Applied Biosystems, Vienna, Austria). We found 16 positive clones that contained microsatellite sequences with at least five tandem repeats. Primer pairs were designed using the software Primer 3 [20] with a product size ranging from 100 to 300 base pairs (bp), primer size from 18 to 22 bp, GC % from 40 to 60, and annealing temperature from 57 to 60 °C.

Polymerase chain reaction (PCR) was performed by screening each primer pair through 10 annealing temperatures (between 46 and 55 °C) for 21 individuals from two different *E. erythropappus* populations. The final volume of each reaction was 15 μl using 30 ng of template DNA added to 12 μl reaction mixture containing 3.33 mM IB Phoneutria buffer (consisting of 100 mM Tris–HCl pH 8.4; 500 mM KCl; 1% Triton X-100; 15 mM MgCl_2_), 1.5 mM MgCl_2_, 0.28 mM of each dNTP, 1 U Taq polymerase, and 0.22 mM of each primer (forward and reverse). The temperature regime was assessed separately for each primer pair; as such, we tested a total of 17 temperatures (from 46 to 62 °C) for six individuals from two populations using MJ Mini™ Thermal Cycler (Bio-Rad, Singapore). The optimal PCR profile used for the amplification of each microsatellite consisted of an initial 3 min at 94 °C, followed by 30 cycles of denaturation at 94 °C for 30 s, annealing temperature (Table 2) for 30 s, extension at 72 °C for 1 min, and a final extension at 72 °C for 7 min. Amplifications were performed using a MJ Mini™ Thermal Cycler.

**Table 2:** Characteristics of nine microsatellite loci developed for *Eremanthus erythropappus* from Minas Gerais State, Brazil

Locus	Primer sequences (5′-3′)	Repeat motif	Size	Tm (°C)	GenBank
ERE02	F: TCTTGCTTACGCGTGTGACT	(GA)21	119	53	MK075833
	R: TGCATCCACTCCAATCACTT				
ERE03	F: GAAGGGAGACATCGGAAGAA	(CTT)5; (CTT)9; (CTT)10; (CTT)8	232	53	MK075834
	R: ACGGAACGGAGAAGAAGAAA				
ERE04	F: CAGTGAGGGGAAGGGAGAAT	(CTT)37	398	53	MK075835
	R: CCTCCACTATAGGGCGGAAT				
ERE07	F: GCGTGGGACTAACCCATTC	(CTT)9	120	53	MK075836
	R: ACCTGTTGGTGAAAGGATGC				
ERE08	F: GAGCCTTCCATGGGAGTAGG	(AGC)5	238	53	MK075837
	R: TGGGAGGGAGAAATTGAACA				
ERE09	F: GCTTACGCGTGGGACTAACT	(CA)3; (GA)8; (GTA)6	269	53	MK075838
	R: GCGTGGACTAGGAAAACGAA				
ERE10	F: GATCATCGCCATGAAGGAAT	(GAA)3; ATA; (GAA)27	244	53	MK075839
	R: CAGTGAGGGGAAGGGAGAAT				
ERE13	F: GAGACCCTGGCTGTCTTCAT	(CT)6; (CA)6	378	53	MK075840
	R: GCGTTGAGTTTCGGAGAAGT				
ERE14	F: CATCGATTTGGAGGCTTCAT	(CT)11; (AT)8; (GT)18	207	53	MK075841
	R: TGCTTACGTGTGCTCTTGCT				

Locus name, primer sequence (F: forward, R: reverse), repeat motif, fragment size (base pair), Temperature of melting (Tm), and GenBank accession numbers are shown

Additionally, we sampled 42 individuals from population 1 (Itamonte: 22°16′45.00″S 44°46′25.60″W) and population 2 (Lavras: 21°19′51.85″S 44°57′54.76″W) to evaluate the SSR primer pairs. Voucher specimens were deposited in the ESAL herbarium of the Federal University of Lavras (UFLA), Brazil. Amplification was conducted using the thermocycler GeneAmp PCR System 9700. We applied the same reaction components and PCR thermal cycle used in the validation process. We separated the PCR products using a 3% high-resolution MetaPhor™ agarose (Lonza, Rockland, Maine, USA) stained with GelRed^→^. Allele sizes were estimated by comparison to a 10-bp DNA Ladder standard (Invitrogen, Carlsbad, California, USA). Individuals that failed to amplify at a minimum of three primer pairs were excluded.

To analyze genotyping errors due to the presence of null alleles, stuttering, and allele dropout, we applied the Brookfield 1 method [2] using 1000 permutations and the Micro-Checker 2.2.3 software [26]. We estimated allele richness using the MSA software [5]. The number of alleles per locus (*N*_*A*_), observed heterozygosity (*H*_*O*_), and expected heterozygosity (*H*_*E*_) for each population and locus according to the Hardy–Weinberg equilibrium were calculated using GenAlEx 6.4 [16]. The within-population inbreeding coefficients (*F*_*IS*_) were determined using FSTAT 2.9.3.2 [8], applying a Bonferroni correction for multiple comparisons. The probability of non-exclusion for each locus, the combined probability of paternity exclusion, and the PIC were calculated using CERVUS 3.0 [9].

We also tested for cross-amplification into two other species, *E. incanus* and *E. glomerulatus.* We amplified the DNA of 10 individuals for each species from one population located in the Beautiful River Falls Ecological Park, Lavras, Minas Gerais State, Brazil (21°19′44.98″S; 44°58′24.58″W). PCR reactions and electrophoresis were performed following the same protocol described above. Subsequently, we compared amplification bands to identify the primers that showed amplification patterns for the two species.

## Data Availability

The datasets used and/or analyzed during the current study are available from the corresponding author on reasonable request.

## Abbreviations

PCR: Polymerase chain reaction
CTAB: Cetyltrimethylammonium bromide
SSR: Simple sequences repeats
PIC: Polymorphic information content
DNA: deoxyribonucleic acid
H_O_: Observed heterozygosity
H_E_: Expected heterozygosity
F_IS_: Inbreeding coefficient
